# Searching for a Relationship between Early Breastfeeding and Cognitive Development of Attention and Working Memory Capacity

**DOI:** 10.3390/brainsci13010053

**Published:** 2022-12-27

**Authors:** Tiziana Pedale, Serena Mastroberardino, Claudia Del Gatto, Michele Capurso, Francesca Bellagamba, Elsa Addessi, Simone Macrì, Valerio Santangelo

**Affiliations:** 1 Department of Physiology and Pharmacology “Vittorio Erspamer”, Sapienza University of Rome, P.le A. Moro 5, 00185 Rome, Italy; 2 Functional Neuroimaging Laboratory, IRCCS Santa Lucia Foundation, Via Ardeatina 306, 00179 Rome, Italy; 3 Department of Philosophy, Social Sciences & Education, University of Perugia, Piazza G. Ermini 1, 06123 Perugia, Italy; 4 Cognitive and Clinical Psychology Laboratory, Department of Human Sciences, Università Europea Di Roma, Via degli Aldobrandeschi, 190, 00163 Rome, Italy; 5 Dipartimento di Psicologia Dinamica, Clinica e Salute, Sapienza Università di Roma, Via degli Apuli 1, 00185 Rome, Italy; 6 Consiglio Nazionale delle Ricerche, Istituto di Scienze e Tecnologie della Cognizione, Via Ulisse Aldrovandi, 16/b, 00197 Rome, Italy; 7 Centre for Behavioural Sciences and Mental Health, Istituto Superiore di Sanità, Viale Regina Elena, 299, 00161 Rome, Italy

**Keywords:** alerting, orienting, conflict, executive function, attention, working memory capacity, breastfeeding, cognitive development

## Abstract

Previous research consistently reported that subjects that were exclusively breastfed (eBF) vs. not-exclusively breastfed (neBF) during infancy (0–6 months) showed increased scores of general intelligence measures (e.g., the intelligence quotient). However, the existent literature largely neglected whether breastfeeding also affects specific cognitive processes, such as attention and working memory (WM) capacity. We tested whether eBF vs. neBF subjects showed performance differences in relation to these two core cognitive functions. The Attention Network Test (ANT), to measure alerting, orienting, and conflict, and the Change Colour Task (CCT), to measure visuospatial WM capacity, were administered to 144 participants divided according to age (6-, 10-, and 18-year-old participants) and breastfeeding (eBF or neBF during 0–6 months of life). Importantly, the sub-groups were homogenous in terms of maternal education, a factor potentially affecting the relation between breastfeeding and cognition. While we found increased performance as a function of participants’ age in both tasks, we failed to observe effects related to breastfeeding, as evidenced by Bayesian analyses. These findings highlight for the first time that the pattern of nutrition provided during early infancy does not appear to affect the development of attention and WM capacity, at least starting from the age considered in the present study.

## 1. Introduction

The attempt to establish a link between cognitive development and nutrition (i.e., breastfeeding vs. formula milk) during the early stages of life (typically ranging from 0 to 6 months) has a long tradition starting from the first decades of the previous century [1]. Since then, several empirical studies further addressed this topic, whose findings have been summarised by numerous systematic reviews (see, e.g., [2,3,4]) that consistently reported an overall increase in measures related to general intelligence (i.e., the Intelligence Quotient, IQ; e.g., [5]) for breastfed children. For instance, Horta and colleagues [4] included in their systematic review 17 studies, showing that exclusively breastfed (eBF) subjects achieved overall an average of 3.44 points higher IQ than not-exclusively breastfed (neBF) subjects. Interestingly, the IQ enhancement—albeit smaller in magnitude—remains significant in studies that controlled for external variables, such as maternal years of formal education, maternal IQ, or maternal/familiar socioeconomic status. As the positive effect of breastfeeding on cognition was also observed in randomised trial studies, the authors concluded that this probably suggests a causal association between breastfeeding and cognition. Some morphometric brain imaging studies, showing volumetric increases in white/grey matter in eBF vs. neBF, appear to support this latter view (e.g., [6,7,8]). These data received further support from prospective studies conducted in preclinical models. Specifically, Hauser and colleagues [9] investigated the long-term consequences of selective deprivation of specific nutritional components of maternal milk during lactation. The authors provided neonate mice with milk devoid of a specific human milk oligosaccharide (6′-Sialyllactose) and demonstrated that this manipulation persistently impaired memory and executive functions in adulthood. Similarly, Pisa and colleagues [10] reported analogous effects in adult mice that, in infancy, received milk with reduced concentrations of a different human milk oligosaccharide (6′-Sialyllactose).

However, it might be worth noting that most of the aforementioned studies are correlational in nature. An inconsistent pattern of findings emerges when considering studies in which the impact of omega-3 fatty acids (as prescribed for a variety of conditions) during pregnancy and/or post-partum was assessed. A recent systematic review on humans failed to report a consistent effect of this kind of supplementation on several outcomes, including children’s cognitive development [11]. Overall, this literature therefore reveals not fully consistent findings that deserve further investigation. Similarly, the few studies that tried to establish a link between the pattern of nutrition provided during early infancy and the development of specific cognitive functions beyond the general IQ score measure provided mixed findings. While the development of some cognitive functions was positively associated with breastfeeding (i.e., language and verbal intelligence), other important functions, such as attention and working memory (WM), were apparently unaffected by the pattern of nutrition in infancy [12,13,14].

In the current study, we specifically aimed at investigating more in depth these two key cognitive processes, attention and WM, and their developmental trajectory as a function of eBF vs. neBF. Importantly, clear developmental trajectories have been documented for both attention and WM (see, e.g., [15,16,17,18,19,20,21,22,23]). These two cognitive processes might be therefore highly sensitive—in principle—to different nutrition patterns at early stages of life. While the above-mentioned literature typically used paper and pencil test batteries, here we examined the impact of breastfeeding on attention and WM using an alternative and innovative approach based on highly sensitive computerised tasks, adapted for children.

Attention was investigated through the Attention Network Test (ANT, [24]). This is a high-sensitivity and widely used test (for a recent meta-analysis, see [25]) that allows characterising three components or networks of attention: the alerting, which involves the capability to react to high-intensity stimuli changing one’s own state of arousal; the orienting, which involves the capability to selectively allocate one’s own attentional resources on a specific stimulus or location; and the executive control, which involves the capability to manage inhibitory control and conflict resolution. Similarly, WM capacity was investigated through another highly sensitive and widely used task, the Change Colour Task (CCT; for a review, see [26]) that allows estimating the amount of information retained in WM.

We compared attention and WM performance of young and older children of 6 and 10 years old, respectively, as previous evidence indicated dramatic developmental changes in both processes across these ages (e.g., [16,20,21]). Children’s performance was then compared to a group of young adults (18 years old). Specifically, both the ANT and the CCT were administered to an overall sample of 144 participants: 50 six-year-old children (mean age = 6.4), 55 ten-year-old children (mean age = 10.2), and 39 young adults (mean age = 18.8 years). Participants were divided into subgroups according to eBF vs. neBF within the first 6 months of life. This information was obtained by asking mothers to report whether the participant was exclusively or not exclusively breastfed during the first 6 months of life. Importantly, we preliminarily assessed the absence of between-group differences of external factors, namely the maternal years of formal education, thus ruling out a potential confound on the participants’ performance in the attention and WM capacity tasks. Resting upon the hypothesis that the mixed findings reported in the available literature [12,13,14] might be due to the limited sensitivity of the test batteries traditionally adopted to evaluate attention and WM, we hypothesised to observe performance enhancement in eBF vs. neBF participants using highly sensitive tests, such as the ANT and CCT.

## 2. Materials and Methods

### 2.1. Participants

A total of 144 healthy participants volunteered for and took part in the study. In Table 1, we reported the participants’ demographic characteristics for each age group (young children, older children, and adults) according to the breastfeeding condition at the early stage of life (0–6 months). The pattern of nutrition was assessed with an ad-hoc questionnaire, in which we asked whether the participant was exclusively or not exclusively breastfed (i.e., exclusively fed with formula milk or a mix of maternal and formula milk) during the first six months of life. For the child participants, the questionnaire was filled in by their mothers, while the adult participants filled in the questionnaire independently, in some cases after contacting their parents to ask for relevant information about the infancy pattern of nutrition. In the questionnaire, we also asked for the maternal years of formal education. Each participant was informed of the experimental procedure and that they could withdraw from the experiment at any time. Parental consent was obtained for each child participant. All adult participants provided informed consent. One young child failed to complete the CCT, while one older child and one adult participant failed to complete the ANT. The children were recruited across three Comprehensive Schools (“Giovan Battista Valente”, Rome, Italy; “Enzo Giuliani” and “Umberto Fifi”, Bastia Umbra, Perugia, Italy), while the adults were recruited from the University of Perugia. The exclusion criteria for children included a diagnosis of neurodevelopmental disorders, as reported by parents or teachers. All participants had a normal or corrected-to-normal vision and were naïve to the main purpose of the study, which was conducted in adherence to the tenets of the Declaration of Helsinki. The study was approved by the independent Ethics Committee of Fondazione Santa Lucia, IRCCS (CE/PROG.665). 

### 2.2. Stimuli and Task

In the current study, we assessed whether early breastfeeding affects attention and WM capacity using the Attention Network Test (ANT; [24]) and the Change Colour Task (CCT; [27]), respectively. Participants sat in a quiet room in front of a laptop computer. The laptop display was placed approximately 50 cm from the viewer (display size = 29° × 22° of visual angle). Participants were also administered a third task—related to the interplay between attention and short-term memory—that was reported elsewhere [21]. The task order was counterbalanced across participants. The presentation of the stimuli was conducted with Cogent 2000 (http://www.vislab.ucl.ac.uk/cogent_2000.php; accessed on 27 October 2017) running on MATLAB 7.1 (The MathWorks Inc., Natick, MA, USA).

### 2.3. ANT

**Figure 1:**
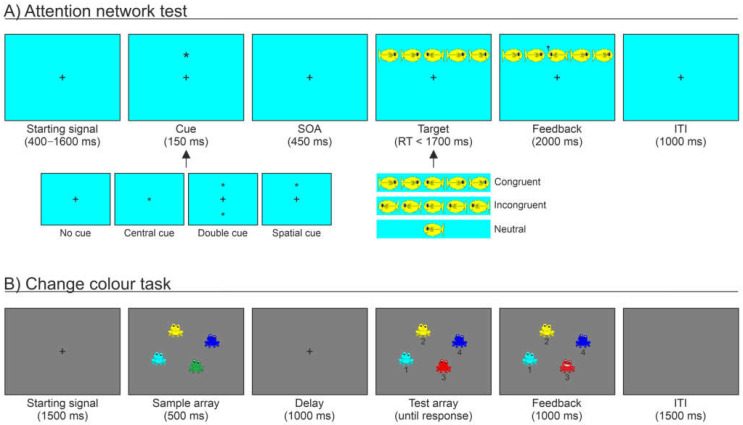
Experimental protocol consisting of the Attention Network Test, ANT (**A**), and Change Colour Task, CCT (**B**). (**A**) Schematic diagram showing the sequence of events during one ANT example trial (adapted with permission from Rueda et al. [22]. Copyright © 2022 Elsevier Ltd.). After a starting signal, a central, double, or spatial cue (i.e., an asterisk), or no cue, was presented. After the SOA, a target was presented, consisting of a central yellow fish oriented either on the left or right side. The target fish could be presented alone (neutral trials) or along with flanking fishes oriented toward the same or opposite direction (congruent or incongruent trials, respectively). Participants discriminated the left vs. right target orientation and then received feedback related to their response, consisting of either the central target fish blowing bubbles and a “Woohoo!” sound (correct response), or a single tone with no animation of the fish (incorrect response). (**B**) Schematic diagram showing the sequence of events during one example trial of the CCT, here adapted for children. After a starting signal, a sample array including a variable set size of 2, 4, or 6 coloured frogs was presented. After a maintenance period, a test array was presented, identical to the sample array, except that one frog always changed in colour. Participants digited on a keyboard the number corresponding to the frog that changed in colour, and then a feedback display was provided, consisting of either a smiling frog replacing the target frog along with a “Woohoo!” sound (correct response), or a single tone with no frog animation (incorrect response).

We used an ANT version adapted for children [22]. Figure 1A illustrates the stimuli and an example trial. Each trial consisted of the presentation of an initial fixation cross (on a cyan background) for a duration ranging from 400 to 1600 ms. After this interval, an attentional cue (i.e., an asterisk) could be presented for 150 ms. The cue could either replace the fixation point (i.e., “central cue” condition), be presented both above and below the fixation point (i.e., “double cue” condition), or be presented either above or below the fixation point (i.e., “spatial cue” condition). Otherwise, no cue was presented (i.e., “no cue” condition). After a fixed stimulus onset asynchrony of 450 ms, in which the fixation cross was presented, a target appeared. This consisted of a central yellow fish (i.e., aligned with the centre of the display) whose face could be oriented either on the left or right side. The target fish could be presented alone (“neutral” trials) or along with flanking fishes oriented toward the same (“congruent” trials) or the opposite direction (“incongruent” trials). Fish could be presented either above or below the central fixation point. The participants were asked to discriminate the left vs. right target orientation by pressing as quickly as possible one of two response keys, within a temporal window of 1700 ms. Then, a feedback display was presented for 2000 ms. Correct responses were followed by the picture presenting the central target fish blowing bubbles and a “Woohoo!” sound, while incorrect responses were followed by a single tone and no animation of the fish. After 1000 ms of inter-stimulus interval, a new trial began. 

During the task, the participants were presented with 96 trials, derived from the combination of the four cue conditions (no cue, central cue, double cue, and spatial cue), two target locations (above vs. below fixation), two target orientations (right vs. left), three target types (congruent, incongruent, and neutral), and two repetitions, for approximately 6.5 minutes. Before starting the task, the participants underwent a practice session of 24 trials. 

### 2.4. CCT

The CCT was modelled after Gold et al. ([27], Experiment 5) and here adapted for children. Figure 1B shows the stimuli and an example trial. Each trial started with a fixation cross with a fixed duration of 1500 ms, with a grey background. A sample array including a variable set size of 2, 4, or 6 coloured frogs (1.36° × 1.36°) was then presented for 500 ms. Each frog was presented in one out of nine possible colours: red, green, blue, yellow, brown, violet, cyan, pink, and orange. The position of each frog was randomly chosen from a set of 25 possible locations defined by dividing the viewing area into a 5 × 5 grid, with a distance between the locations of 3.12°. After a delay of 1000 ms, a test array was presented. This was identical to the sample array, except that one frog always changed to a new colour, and that under each frog was presented a digit number (from 1 to 6, according to the current set size). The participant’s task was to digit on the keyboard the number corresponding to the frog that changed in colour. The test array was displayed for an unlimited amount of time until the participant provided a response. Participants were instructed to take their time and to be as accurate as possible. After a response was provided, a feedback display was presented for 1000 ms. For correct responses, a smiling frog replaced the frog that was correctly identified as changing in colour, along with a “Woohoo!” sound; for incorrect responses, a single tone was presented with no animation of the frog. After 1500 ms of inter-stimulus interval, a new trial began.

The participants completed 72 trials, i.e., 24 repetitions for each set size, for a duration that lasted usually no longer than 10 minutes. Before starting the task, participants underwent a practice session of 12 trials.

### 2.5. Data Analysis

Before analysing the data derived from the ANT and CCT, we conducted a preliminary check to assess whether the level of formal education (in years) of participants’ mothers did or did not differ between eBF and neBF participants in the three age groups.

Then, to analyse the data derived from the ANT, we computed three measures related to alerting, orienting, and conflict (cf. [22,24]). Firstly, we computed the participants’ median reaction times (RTs) for each cue condition (across the three target types), and for each target type (across the four cue conditions). Then, alerting was computed by subtracting median RTs for “no cue”−median RTs for “double cue” trials; orienting was computed by subtracting median RTs for “central cue”−median RTs for “spatial cue” trials; finally, conflict was computed by subtracting median RTs for “incongruent”−median RTs for “congruent” trials. For each measure, the lower the score, the higher the efficiency in measured component (i.e., alerting, orienting, and conflict control).

For the data derived from the CCT, participants’ performance was measured using the Pashler/Cowan K equation, where K represents how many items worth of information have been stored in WM [28,29]. Following Gold et al. [27], we computed the K score using the formula: K = (proportion correct × set size) − 1, with a maximum K score for a given set size equal to set size − 1.

At the statistical level, two-way Bayesian analyses of variance (ANOVAs; [30]) were used to evaluate whether the attentional components derived from the ANT task (i.e., alerting, orienting, and conflict) varied as a function of the between-participants factor of age group (3 levels: young children, older children, or adults), and the between-participants factor of breastfeeding (2 levels: eBF, and neBF). A three-way mixed Bayesian ANOVA [30] was used to evaluate whether the WM capacity score derived from the CCT (i.e., the K score) varied as a function of the within-participants factor of set size (3 levels: 2, 4, or 6 coloured frogs to remember), the between-participants factor of age group (3 levels: young children, older children, or adults), and the between-participants factor of breastfeeding (2 levels: eBF and neBF). 

The advantage of the Bayesian approach is that, while classical frequentist ANOVA allows only to accept or reject the presence of an effect, Bayesian ANOVA offers the possibility to select the best model (BF_M_) and, through model comparisons, allows to evaluate the strength of evidence in favour (BF_10_) or against (BF_01_) the inclusion of each factor according to the following interpretation: (BF) < 1: no evidence, 1–3: anecdotal evidence, 3–10: substantial evidence, 10–30: strong evidence, 30–100: very strong evidence, >100: decisive evidence [31,32]. The strength of evidence in favour or against the inclusion of each factor was estimated by comparing the model containing the effect of interest with the matched model stripped of the effect. For post hoc tests, correction for multiple comparisons was performed using the approach discussed in Westfall [33], which consists of the following steps. As a first step, Bayesian t-tests are computed for all pairwise comparisons, thus providing unadjusted Bayes factors (BF_U_); as a second step, the prior model odds are adjusted by fixing the overall probability of no effect to 0.5; finally, the adjusted prior odds and the Bayes factor are used to calculate the adjusted posterior odds. All the statistical analyses were performed using JASP (Version 0.16.4, https://jasp-stats.org/; accessed on 15 June 2021).

## 3. Results

Preliminarily, to rule out the possibility that the participants’ performance at the ANT and CCT was confounded by the maternal level of formal education of participants’ mothers (see [34]), we performed a Bayesian ANOVA on maternal years of formal education to ensure that there was no difference between eBF and neBF participants in the three age groups (see Table 2). This analysis revealed substantial evidence against differences in the maternal level of education between the eBF and neBF participants (BF_01_ = 3.97), as well as substantial evidence against an age group by breastfeeding interaction (BF_01_ = 7.51). These results confirmed that mothers’ education did not differ between eBF and neBF participants in the three age groups.

Having established that there were no differences in the years of maternal formal education among the different sub-groups, we asked whether the three attentional components derived from the ANT (i.e., alerting, orienting, and conflict) and the WM capacity derived from the CCT (i.e., the K score) were affected by participants’ age and breastfeeding during infancy.

### 3.1. ANT Data

**Figure 2:**
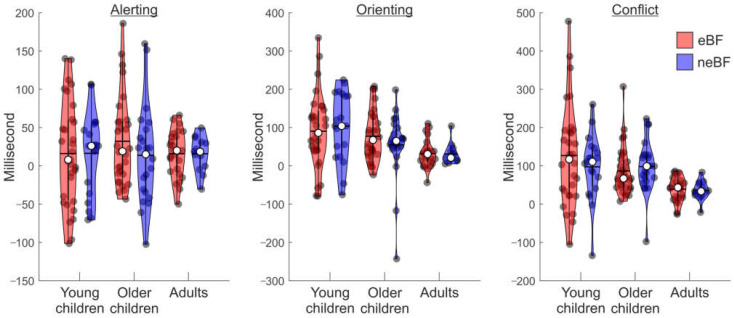
Violin graphs displaying the participants’ performance related to the three components of the ANT, namely alerting, orienting, and conflict, as a function of the age groups, and pattern of nutrition during 0–6 months of life. For each violin, the dark circles represent the individual performance; the median and mean of the distribution are represented by the white circle and the horizontal bar, respectively.

The two-way Bayesian ANOVA on the alerting component revealed as a winning model the null model containing no effect of interest (BF_M_ = 11.25; see Table 3 and Figure 2, left panel). The Bayesian model comparison showed strong evidence against the inclusion of the age factor (BF_01_ = 10.31), as well as substantial evidence against the inclusion of the breastfeeding factor (BF_01_ = 4.32), and the age group by breastfeeding interaction (BF_01_ = 5.20), thus indicating equal alerting mechanisms in the three groups of age, regardless of the type of nutrition received during the first six months of life.

The two-way Bayesian ANOVA on the orienting component revealed as a winning model the one containing only the age group effect (BF_M_ = 15.73; see Table 3 and Figure 2, central panel). Consistently, the Bayesian model comparison showed very strong evidence in favour of the inclusion of the age factor (BF_10_ = 48.67), while the strengths of evidence against the inclusion of the breastfeeding factor (BF_01_ = 5.31) and the age group by breastfeeding interaction (BF_01_ = 4.51) were both substantial. Post hoc analysis revealed decisive evidence for a lower orienting score (i.e., greater attentional orienting efficiency) in the adult group compared to the young children group (BF_10, U_ = 177.71, posterior odds = 104.39), and substantial evidence for a lower orienting score in the adult group as compared to the older children group (BF_10, U_ = 5.96, posterior odds = 3.50). There was no evidence for differences in the orienting components between the young and older children groups (BF_10, U_ = 0.73, posterior odds = 0.43). Overall, these results indicate a gradual improvement in the orienting component of attention across age, which was not affected by the type of milk feeding received during infancy.

Similar results were obtained on the conflict measure where the two-way Bayesian ANOVA revealed again as the winning model the one containing solely the age group effect (BF_M_ = 15.78; see Table 3 and Figure 2, right panel), with decisive evidence in favour of the inclusion of the age factor (BF_10_ = 252.47), while the strengths of evidence against the inclusion of the breastfeeding factor (BF_01_ = 5.02) and the age group by breastfeeding interaction (BF_01_ = 4.02) were both substantial. Concerning the effect of age, the post hoc analysis revealed decisive evidence for a lower effect of the conflicting cue in the adult group as compared to the young children group (BF_10,U_ = 173.25, posterior odds = 101.77) and to the older children group (BF_10,U_ > 1000, posterior odds = 1349.21). There was no evidence for differences in the conflict component between the young and older children groups (BF_10,U_ = 0.45, posterior odds = 0.26). These results indicate that the control of conflicting stimuli also improves across age and that this ability appears not to be affected by the type of milk feeding received during infancy.

### 3.2. CCT Data

**Figure 3:**
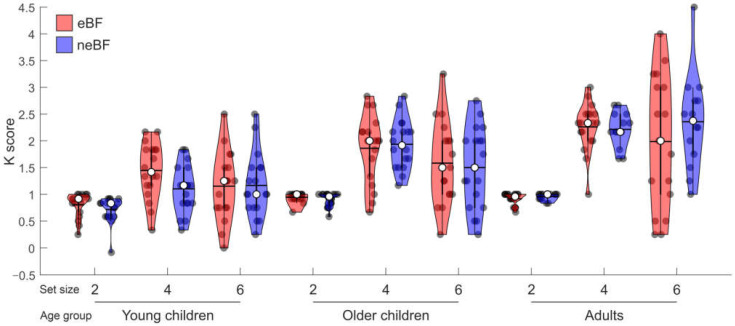
Violin graphs displaying the participants’ performance at the CCT as a function of the set sizes, age groups, and pattern of nutrition during 0–6 months of life. For each violin, the dark circles represent the individual performance; the median and mean of the distribution are represented by the white circle and the horizontal bar, respectively.

Concerning the WM capacity measures, the three-way mixed Bayesian ANOVA on the K score derived from the CCT revealed as the winning model that containing the age group and set size factors, and their interaction (BF_M_ = 79.47; see Table 4 and Figure 3), thus indicating that the breastfeeding factor did not affect the long-term development of WM capacity. Specifically, the evidence in favour of the inclusion of the age group, set size, and their interaction was decisive (all BF_10_ > 1000), while the evidence against the inclusion of the breastfeeding factor (BF_01_ = 5.90), and its interaction with the age factor (BF_01_ = 5.33) and the set size (BF_01_ = 7.92) were substantial. Strong was also the evidence against the inclusion of the three-way interaction between set size, age, and breastfeeding (BF_01_ = 17.96). The post hoc analysis revealed strong evidence supporting increased WM capacity in the adult group as compared to the older children group (BF_10,U_ = 14.61, posterior odds = 8.58), who, in turn, showed decisive evidence for a higher WM capacity as compared to the young children group (BF_10,U_ > 1000, posterior odds = 57,153.24). As regards to the set size, the post hoc analysis revealed, with substantial evidence, a higher K score when the to-be-remembered items were 6 compared to 4 (BF_10,U_ = 4.23, posterior odds = 2.49), and, with decisive evidence, a higher K score when the to-be-remembered items were 4 compared to 2 (BF_10,U_ > 1000, posterior odds = 7.76 × 10^33^). Finally, the post hoc analysis on the interaction revealed that all age groups showed with decisive evidence a higher K score with a set size of 4 compared to 2 (BF_10,U_ > 1000, posterior odds = 857.76 for the group of younger children; BF_10,U_ > 1000, posterior odds = 5.63 × 10^15^ for the group of older children; BF_10,U_ > 1000, posterior odds = 5.34 × 10^24^ for the group of adults). On the contrary, only the older children showed substantial evidence for a higher K score with a set size of 4 compared to 6 (BF_10,U_ = 3.55, posterior odds = 0.59), while no evidence was revealed in the other two groups of age between the set size of 4 and 6 (BF_10,U_ = 0.26, posterior odds = 0.04 for the young children, and BF_10,U_ = 0.25, posterior odds = 0.04 for the adults).

## 4. Discussion

The current study aimed at investigating the potential impact of breastfeeding (eBF vs. neBF) at the early stage of life (0–6 months) on two core cognitive functions, attention and WM capacity, as measured at 6, 10, and 18 years old. These functions were assessed using two well-established and reliable tasks, the ANT and the CCT. We found that the attentional indexes (except for the alerting; see below) as well as the WM capacity indexes improved as a function of age. However, we failed to observe any effect of breastfeeding on performance modulation.

Regarding the attention indexes measured by the ANT, both attentional orienting and conflict control improved as a function of age, as evidenced by the Bayesian analyses showing that the inclusion of the age factor greatly improved the predictive performance of both models. Concerning the conflict index, which is a measure of executive control that involves the capability to inhibit inappropriate responses that might arise from conflicting information [24,35], the literature is consistent in showing a clear developmental trajectory from childhood to adulthood (as observed in the current study), both using the ANT (e.g., [17,22,36,37]) and other measures (see, e.g., [16,20]; for recent reviews, see [38,39]). In contrast, the literature on the development of attentional orienting is mixed: some pieces of evidence support a developmental invariance for attentional orienting (e.g., [22,36]), while other findings revealed a developmental gradient from childhood to adulthood in the deployment of endogenous (i.e., voluntary) spatial attention, that is, the specific type of attentional orienting implemented in the ANT (see, e.g., [16,20,40,41,42]). The current findings corroborate this latter view, supporting a gradual development in the efficiency of allocating visuospatial attentional resources from early to late childhood, and then adulthood. 

The current findings support, instead, developmental invariance in relation to the alerting index, for which the Bayesian model showed strong evidence against the inclusion of the age factor to account for the data distribution. Although there is evidence, derived from the ANT, of alerting modulations as a function of age (e.g., [17,37,43]), there is also contrasting evidence that supports developmental invariance of alertness, for instance, in young children (i.e., 4–6 years old, [44]), but also across extended developmental stages (i.e., in 4–13-year-old children compared to young adults; [45]). Likewise, we found a substantial similarity in the alertness across the three age groups, although it might be worth noting here the larger variability in the data distribution for children as compared to adults (as indicated by the distribution of dark dots in the first four violin plots vs. the last two violin plots in Figure 2, left panel).

As regards to visuospatial WM capacity, here measured through the CCT, our findings are overall consistent with the extant literature, as developmental studies have repeatedly documented that mature WM capacity is only reached during late childhood or adolescence. Interestingly, there is an ongoing debate as regards to the developmental turning point. Some studies report mature visuospatial WM capacity around 10–12 years of age [46,47], while other studies report that mature WM capacity is reached only later, around the age of 16 [23,48,49,50]. The current findings are in line with this latter view. The Bayesian analyses revealed strong evidence in support of increased WM capacity in the adult group as compared to the older children group, who, in turn, showed decisive evidence for a higher WM capacity as compared to the young children group, overall suggesting that 9–11 years of age are not enough to guarantee a fully mature visuospatial WM capacity. Incidentally, here we offered a new adaptation of the classical CCT, namely a version adapted for children, that might be useful to promote further research at the developmental level.

Consistently with the main aim of the current study, here we assessed whether attention and WM age-related performance varied as a function of the feeding pattern received at early stages of life (0–6 months). Notwithstanding the developmental trajectories observed for both the ANT and the CCT, we failed to observe a modulation of the performance at the ANT and CCT as a function of breastfeeding (eBF vs. neBF). The Bayesian analyses showed clear evidence against the inclusion of the breastfeeding factor to improve the predictability of the model, both in the ANT and CCT. Notwithstanding the use of high-sensitivity computerised tasks to measure attention and WM capacity, these findings are overall consistent with the previous investigations, which showed an impact of breastfeeding on the development of language and verbal intelligence, but not on the cognitive functions here under investigation (i.e., including attention and WM) [12,13,14].

Interestingly, we did not observe any difference between eBF and neBF sub-groups of participants in terms of maternal years of formal education, which may be pivotal for the interpretation of the current findings. A recent meta-analysis conducted by Mohammed and colleagues [51] asked whether the association between breastfeeding and enhanced cognition might reflect the socioeconomic status of those who breastfed and the related family. To this aim, these authors carried out a meta-analysis of those studies that investigated the link between breastfeeding and cognitive development in sub-Saharan African populations, where breastfeeding is—typically—the norm, and therefore not correlated with the maternal/family socioeconomic status. None of the 17 studies included in the analysis found a reliable association between breastfeeding and cognitive development in children or adolescents in sub-Saharan Africa. Mohammed and colleagues interpreted these findings as evidence that breastfeeding does not necessarily improve cognitive development, which might be instead a consequence of external variables, such as those related to socioeconomic status. Consistently with this meta-analysis, here we showed no impact of breastfeeding in sub-groups of participants that did not differ in terms of the maternal level of formal education. Future research might be conducted to disentangle the specific role played by the co-occurrence of external/socioeconomic factors and breastfeeding to determine the developmental trajectories of attention and WM capacity.

It might be worth noting at this point some limitations of the current study. Here we focused on two specific age groups of 6- and 10-year-old children; considering a larger spectrum of ages—especially younger children—would allow gleaning a more complete picture of the potential impact of breastfeeding on attention and WM capacity. In fact, it is possible that any difference between eBF and neBF extinguished by the age of 6 years old. Similarly, in the current study, we characterised attention and WM capacity using two specific tasks, the ANT and CCT. Although these are two validated and reliable measures, it is possible that other measures of attention and WM capacity might be more sensitive to highlight a modulation of breastfeeding, for instance, measures integrated with daily school activities (see, e.g., [52]).

Moreover, it should be considered that the current study, yielding negative results, focused only on the difference between exclusively breastfed infants and not exclusively breastfed infants. In this research area, the comparison between studies is complicated by the fact that there is a mixture of feeding methods (direct milk at the breast, pumped milk through a bottle, donor human milk, and a combination of these methods). Some of the studies documenting positive effect of breastfeeding on cognitive development compared exclusively breastfed infants with those exclusively fed with formula milk [53,54] or considered the impact of the duration of breastfeeding even beyond the first 6 months of life [55]. Moreover, we have here considered a dichotomous measure of breastfeeding pattern (eBF vs. neBF) that does not consider the actual proportion of maternal milk vs. formula taken in by the child in the neBF group. Finally, it is important to note that we assessed breastfeeding using a retrospective maternal self-report measure. Even if we can assume that mothers are reliable when reporting on such an important and emotionally salient aspect of their relationship with their offspring, as the personal history of breastfeeding [56], still we cannot exclude that individual differences may exist between mothers in their ability to accurately recollect about a feeding practice that had occurred from 6 to 18 years before the time of their report.

Notwithstanding these limitations, which should be desirably addressed by future research, the current findings highlight for the first time that the pattern of nutrition provided between 0 and 6 months of infancy does not appear to systematically affect attention and WM capacity (as measured in terms of ANT and CCT), at least starting from the developmental stage here taken into account, i.e., 6 years of age. 

The available scientific evidence for long-term effects of breastfeeding on cognition is still debated. Given the importance of breastfeeding from a public health and clinical perspective [57], it is impelling the need for more longitudinal studies and researches based on cluster-randomised trials to evaluate the benefits of breastfeeding throughout life.

## Figures and Tables

**Table 1:** Participants’ demographic characteristics for each age group.

	No. of Participants	Gender		Age	
		Males	Females	Mean ± S.D.	Range
Young Children	50	19	31	6.4 ± 0.5	6–7
eBF	31	13	18	6.4 ± 0.5	6–7
neBF	19	6	13	6.3 ± 0.5	6–7
Older Children	55	28	27	10.2 ± 0.4	9–11
eBF	32	17	15	10.2 ± 0.4	10–11
neBF	23	11	12	10.2 ± 0.5	9–11
Adults	39	14	25	18.8 ± 0.9	17–20
eBF	25	11	14	18.8 ± 0.9	17–20
neBF	14	3	11	18.8 ± 0.9	17–20

**Table 2:** Analysis of the effects in the Bayesian ANOVA conducted on maternal years of formal education.

Predictors	P(incl)	P(excl)	P(incl|data)	P(excl|data)	BF_01_	BF_10_
Age	0.400	0.400	0.503	0.483	0.960	1.042
Breastfeeding	0.400	0.400	0.198	0.788	3.974	0.252
Age *x* breastfeeding	0.200	0.200	0.014	0.103	7.511	0.133

P(incl) and P(excl) represent the priors of including and excluding the predictor before seeing the model. P(incl|data) and P(excl|data) represent the posteriors of including and excluding the predictor after seeing the model. BF_01_ represents how much more likely the data are under the model excluding the predictor than under the model including the predictor, while BF_10_ represents how much more likely the data are under the model including the predictor than under the model excluding the predictor.

**Table 3:** Analysis of the effects in the Bayesian ANOVAs conducted on the measures of alerting, orienting, and conflict, derived by the ANT.

Alerting						
Predictors	P(incl)	P(excl)	P(incl|data)	P(excl|data)	BF_01_	BF_10_
Age	0.400	0.400	0.088	0.909	10.314	0.097
Breastfeeding	0.400	0.400	0.187	0.810	4.323	0.231
Age *x* breastfeeding	0.200	0.200	0.003	0.016	5.201	0.192
**Orienting**						
**Predictors**	**P(incl)**	**P(excl)**	**P(incl|data)**	**P(excl|data)**	**BF_01_**	**BF_10_**
Age	0.400	0.400	0.947	0.019	0.021	48.666
Breastfeeding	0.400	0.400	0.153	0.814	5.314	0.188
Age *x* breastfeeding	0.200	0.200	0.033	0.150	4.513	0.222
**Conflict**						
**Predictors**	**P(incl)**	**P(excl)**	**P(incl|data)**	**P(excl|data)**	**BF_01_**	**BF_10_**
Age	0.400	0.400	0.957	0.004	0.004	252.473
Breastfeeding	0.400	0.400	0.160	0.801	5.021	0.199
Age *x* breastfeeding	0.200	0.200	0.039	0.159	4.025	0.248

P(incl) and P(excl) represent the priors of including and excluding the predictor before seeing the model. P(incl|data) and P(excl|data) represent the posteriors of including and excluding the predictor after seeing the model. BF_01_ represents how much more likely the data are under the model excluding the predictor than under the model including the predictor, while BF_10_ represents how much more likely the data are under the model including the predictor than under the model excluding the predictor.

**Table 4:** Analysis of the effects in the Bayesian ANOVA conducted on the data derived by the CCT.

Predictors	P(incl)	P(excl)	P(incl|data)	P(excl|data)	BF_01_	BF_10_
Set size	0.263	0.263	1.68 × 10^−6^	1.90 × 10^−41^	1.13 × 10^−35^	8.85 × 10^34^
Age	0.263	0.263	1.66 × 10^−6^	8.52 × 10^−15^	5.12 × 10^−9^	1.95 × 10^8^
Breastfeeding	0.263	0.263	0.138	0.815	5.896	0.170
Set size *x* age	0.263	0.263	1.000	1.71 × 10^−6^	1.71 × 10^−6^	585716.102
Set size *x* breastfeeding	0.263	0.263	0.021	0.164	7.917	0.126
Age *x* breastfeeding	0.263	0.263	0.029	0.155	5.329	0.188
Set size *x* age *x* breastfeeding	0.053	0.053	2.04 × 10^−4^	0.004	17.962	0.056

P(incl) and P(excl) represent the priors of including and excluding the predictor before seeing the model. P(incl|data) and P(excl|data) represent the posteriors of including and excluding the predictor after seeing the model. BF_01_ represents how much more likely the data are under the model excluding the predictor than under the model including the predictor, while BF_10_ represents how much more likely the data are under the model including the predictor than under the model excluding the predictor.

## Data Availability

The dataset generated and analysed during the current study is available from the corresponding author on reasonable request.
